# Manual Compression of the Heart in Syncope Due to Anæsthetics

**Published:** 1902-12-27

**Authors:** 


### MANUAL COMPRESSION OF THE HEART IN SYNCOPE DUE TO ANÆSTHETICS.

A couple of years or so ago we referred to an extraordinary case, published in a Dutch journal, in which, after cessation of the pulse and respiration during the administration of chloroform, and after failure of the methods usually adopted with the object of restoring these functions, the heart was exposed and rhythmically compressed while artificial respiration was carried on, with the result that in the end spontaneous cardiac movements were set up and the patient was restored. It is not every surgeon who would be prepared to undertake such a manœuvre, nor is it in every case that he would be justified in so doing. When, however, syncope occurs in the course of a laparotomy, when the abdomen is already not only prepared for operation but is actually laid open, so that an easy route already exists by which the hand can reach such a position in relation to the heart as to be able to bring effective pressure to bear upon it, the case is very different, and we can hardly doubt the advantage, under such circumstances, of bringing to bear upon the paralysed heart such rhythmic compression as shall asssist in emptying its overloaded cavities. At a recent meeting (November 7th) of the Society of Anæsthetists, Dr. E. A. Starling read a report of a case in which the above-mentioned proceeding was adopted. The patient was a man, 65 years of age, on whom an operation for adhesions about the colon was being performed by Mr. Arbuthnot Lane. Ether preceded by gas was the anæsthetic which was used. The induction of anæsthesia was easy and natural, but after the appendix had been removed the pulse began to flag, the respiration which had been shallow not being then affected. More ether was given, but while the stump was being trimmed both pulse and respiration ceased. Artificial respiration was started but had no good effect. Mr. Lane then introduced his hand through the abdominal incision, and finding that the heart, as felt through the diaphragm, was quite motionless, he gave it a squeeze or two when he felt it start beating again. Respiration, however, was still suspended, so artificial respiration was continued for about twelve minutes, when natural respiration recommenced. Except for some pain about the diaphragm for a couple of days the patient made a good recovery. In commenting upon the case Dr. Starling is reported as describing it as one of successful treatment by "direct stimulation of the heart." One cannot but feel, however, that the whole interest of the case hinges upon the fact that it was something more than "stimulation" which set the heart agoing. If the case is to be regarded as one of real importance it is because of the "squeeze or two" given by Mr. Lane—the direct compression of the loaded heart which was sufficient to relieve it of its burden, and to bring the overstretched muscle once more within its range of contractility.

